# Elective single embryo transfer in in vitro fertilization cycles with or without preimplantation genetic testing using next-generation sequencing: A randomized clinical trial

**DOI:** 10.1016/j.clinsp.2026.100870

**Published:** 2026-02-24

**Authors:** Pedro Felipe Magalhães Peregrino, Carlos Augusto Z. Nissel, Patricia Florido, Mayara Satiko Nakano, Marcos de Lorenzo Messina, José Maria Soares Júnior, Edmund Chada Baracat, Pedro Augusto Araujo Monteleone

**Affiliations:** Disciplina de Ginecologia, Departamento de Obstetrícia e Ginecologia, Hospital das Clínicas da Faculdade de Medicina da Faculdade de Medicina da Universidade de São Paulo, São Paulo, SP, Brazil

**Keywords:** Reproductive medicine, In vitro fertilization, Single embryo transfer pregnancy rate, Pre-implantation genetic diagnosis, Next-generation sequencing

## Abstract

**Introduction:**

Preimplantation Genetic Testing (PGT-A) has been increasingly used in IVF to improve embryo selection. However, its clinical utility in patients with a good prognosis remains debated. There is a lack of robust scientific evidence about the benefits of preimplantation genetic testing Next-Generation Sequencing (NGS) on the clinical pregnancy and live birth rates in good-prognosis IVF patients undergoing elective Single Embryo Transfer (eSET) testing.

**Objective:**

To compare the clinical pregnancy rate after IVF treatment in patients with a good prognosis between groups that receive the transfer of a single embryo with or without pre-implantation genetic embryo analysis.

**Methods:**

This work was a prospective randomized clinical study that evaluated 206 patients to compare the clinical pregnancy rate after the first elective transfer of an embryo, associated or not with pre-implantation genetic embryo analysis using Next-Generation Sequencing (NGS). Patients were randomized into groups: SET group ‒ patients undergoing the transfer of a single embryo (eSET, elective Single Embryo Transfer) without embryo genetic analysis (SET group, *n* = 103); and NGS + SET group ‒ patients undergoing eSET with embryo genetic analysis (NGS + SET group, *n* = 103).

**Results:**

The clinical pregnancy rate after the first transfer was similar between the two groups (SET: 76.7 % and NGS + SET: 79.6 %; *p* = 0.613). There was also no statistical difference in live birth rates or abortion rates between the groups analyzed.

**Conclusion:**

This clinical trial suggests no significant improvement in the embryo genetic analysis in IVF treatments for infertile couples with a good prognosis

## Highlights

• Infertility affects around 15 % of couples around the world.
• The quality of eggs and the chances of pregnancy in IVF treatment decrease with maternal age.
• Preimplantation genetic diagnosis can help select euploid embryos for embryo transfer.
• The indication of preimplantation genetic diagnosis in IVF treatment does not seem to reduce treatment costs.
• Preimplantation genetic diagnosis does not seem to ALTER miscarriage or pregnancy rates in good prognosis fertile couples.

## Introduction

Infertility is a significant public health concern, affecting 8–12 % of couples of reproductive age and resulting in a high prevalence of childless couples.^1^^,^^2^ Recent estimates reveal that over 1.8 million Assisted Reproductive Technology (ART) treatments were performed in 2010, leading to the conception of more than 6 million children using ART.^3^ In Brazil, according to the 14th report of the national embryo production system (SisEmbryo), more than 45,000 In Vitro Fertilization (IVF) cycles were performed in 2021.^4^

Historically, it is known that only a limited number of retrieved oocytes have the potential to generate a full-term pregnancy. Thus, embryo selection is a crucial step for the success of IVF techniques. The efficiency of this process is linked to the reduction in the number of embryos to be transferred. Selecting embryos with higher implantation potential makes transferring a lower number of embryos more viable and effective. In routine practice, embryo selection is based on morphological criteria and embryo development characteristics to infer their implantation potential.^5^

Initially, PGS, or preimplantation genetic screening, was performed using *in situ* hybridization technology from a day-3 embryo biopsy, analyzing one or two blastomeres for a limited number of chromosomes. Technological advancements led to the complete analysis of all 24 chromosomes using Comparative Genome Hybridization (CGH) associated with blastocyst-stage embryo biopsy. This approach evaluates a higher number of cells extracted from the trophectoderm. Some studies suggest that this technique is associated with higher clinical pregnancy rates, improved embryo selection in elective Single Embryo Transfer (SET) cycles, while maintaining high pregnancy rates and reducing multiple pregnancies in patients with a good prognosis.^6^ More recently, Next-Generation Sequencing (NGS) has been used and validated for embryo genetic analysis in IVF cycles. It involves Whole Genome Amplification (WGA) and has the potential to improve chromosomal embryo diagnosis in terms of robustness, automation, and the ability to detect aneuploidies.^7^^,^^8^

Classic indications for performing PGS include advanced maternal age,^9^ repeated implantation failures,^10^^,^^11^ recurrent miscarriage,^12^ and severe male factor infertility.^13^ Recently, PGS has been proposed for embryo selection in SET cycles for patients of both young and advanced maternal age, with some studies demonstrating a significant increase in live birth rates per embryo transfer and a reduction in multiple pregnancies.^14^, ^15^, ^16^

Considering that approximately 50 % of embryos exhibit chromosomal abnormalities after controlled ovarian stimulation,^17^ genetic embryo evaluation has become increasingly utilized for selecting euploid embryos for transfer. Recent evidence demonstrates that blastocyst biopsy associated with chromosomal screening is highly predictive of embryo developmental potential. This approach enhances implantation chances by selecting euploid blastocysts for transfer in fresh^18^ and vitrified cycles.^15^

The literature remains controversial regarding the universal application of genetic analysis in all infertile couples and the actual benefits for patients. Questions persist about the necessity of genetic embryo analysis in cases without risk factors and with a favorable prognosis for IVF success. Recent randomized clinical trials, such as the study conducted by Mune et al. in 2019, compared women aged 25‒40 undergoing IVF with NGS to those without embryo biopsy, revealing a statistically significant difference in ongoing pregnancy rates in women aged 35–40.^19^

In the last 10-years, several studies have been published with the aim of demonstrating the increased success rate in IVF treatment through Preimplantation Genetic Diagnosis (PGD). Until 2017, these studies had small study groups, and the literature required greater evidence regarding the application of this technology.

After 2018, more robust studies with greater scientific impact began to emerge. Ozgur et al., also in a randomized clinical trial, included 220 infertile patients under 35-years-old, randomized into two groups: patients undergoing IVF with preimplantation genetic analysis and another without embryo biopsy. Upon analyzing the results, it showed no statistically significant difference in live birth rates (56.3 % in the biopsy group vs. 58.6 % in the non-biopsy group).^20^

Another more recent randomized clinical trial, Yan et al., analyzed 1212 infertile women aged between 20 and 37 undergoing IVF, separated into a group with embryo biopsy and another without genetic analysis. Live birth rates were similar between the two groups and the result demonstrated non-inferiority in cumulative pregnancy rates between the two groups (77.2 % in the genetic analysis group and 81.8 % in the non-biopsy group).^21^

The notable technological advancement in Assisted Reproductive Techniques (ART) is evident. However, in addition to appropriate medical indications, it is necessary to establish and communicate to patients the cost-benefit ratio and the potential for time gain or loss until achieving pregnancy. Perhaps, in many cases, the use of a technology that comes with higher costs may not be necessary without clear evidence of clinical pregnancy rate benefits. This analysis is especially important, particularly in developing countries like Brazil, where there are existing human reproduction centers funded by the government. Some studies suggest that the added value to IVF treatment with biopsy can exceed an increase of over $6000.^22^ However, some works show that preimplantation genetic analysis is financially advantageous in specific cases.^22^, ^23^, ^24^

Especially for women above 37-years-old, the selection of euploid embryos, although not increasing the cumulative pregnancy rate, can reduce the time for the couple to achieve the goal of having a child, in addition to decreasing costs associated with potential implantation failures and abortions.^23^^,^^24^

Engaged in the current literature, which suggests that genetic analysis may not be necessary for patients with a good prognosis and may not result in higher pregnancy rates, there arose the need to assess the real impact of NGS indication on IVF pregnancy rates, within a Brazilian context, in a center where all treatment costs are subsidized by the government. Thus, the authors proposed this study to compare the outcomes of IVF treatments in patients who underwent elective Single Embryo Transfer (eSET) genetically assessed by NGS with a group of patients subjected to eSET without genetic analysis. This approach will confirm or refute the hypothesis that genetic analysis is not effective in improving embryo selection and, primarily, whether its association with eSET can increase clinical pregnancy and live birth rates while reducing abortion rates.

## Methods

### Study design

This is a prospective randomized clinical trial and the CONSORT reporting guidelines.^25^ This study was conducted at a single center to compare the effectiveness of preimplantation embryonic genetic analysis using Next-Generation Sequencing (NGS) in In Vitro Fertilization (IVF) cycles for patients with good prognosis and undergoing elective single embryo transfer.

This study is part of a research project conducted at the Governor Mário Covas Human Reproduction Center within the Gynecology Division of the Hospital das Clínicas at the Faculty of Medicine of the University of São Paulo (HC-FMUSP), registered on ClinicalTrials.gov with the registration number NCT03758833.

### Patients

Between January 2019 and 2021, couples enrolled in a research protocol enrolled at the Mário Covas Human Reproduction Center were selected. They underwent basic clinical investigation, with routine complementary exams for infertility diagnosis. Those with an indication for In Vitro Fertilization (IVF) cycles and meeting the inclusion criteria, while not meeting the exclusion criteria for this study, were invited to participate. All infertile couples with an indication for IVF who agreed to participate in this study signed the Informed Consent Form (ICF) before the start of the proposed treatment.

Inclusion criteria for ovarian stimulation Initiation:

• Couples undergoing high-complexity in vitro fertilization cycles, utilizing Intracytoplasmic Sperm Injection (ICSI);
• Couples undergoing their first treatment cycle;
• Woman's age between 18- and 37-years;
• Body Mass Index (BMI) between 18 and 30 kg/m^2^;
• Presence of both ovaries without evidence of significant abnormalities on pelvic ultrasound;
• Absence of any significant affection of the endometrial cavity, such as polyps, fibroids larger than 4 cm, or hydrosalpinx;
• Adequate ovarian reserve, determined by antral follicle count >8 and FSH levels <12 mIU/mL;
• Use of freshly ejaculated or cryopreserved seminal sample from the partner.
Exclusion criteria for ovarian stimulation initiation:
• Women presenting with endometriosis grades III and IV, according to the classification of the American Society for Reproductive Medicine (ASRM, 2020);
• Use of sperm for fertilization from a partner with severe oligozoospermia (<5 million sperm/mL);
• Women with associated systemic diseases or infectious diseases;
• Non-selection criteria for randomization;
• Lack of at least two good-quality blastocysts on the fifth day of development.

**Fig. 1:**
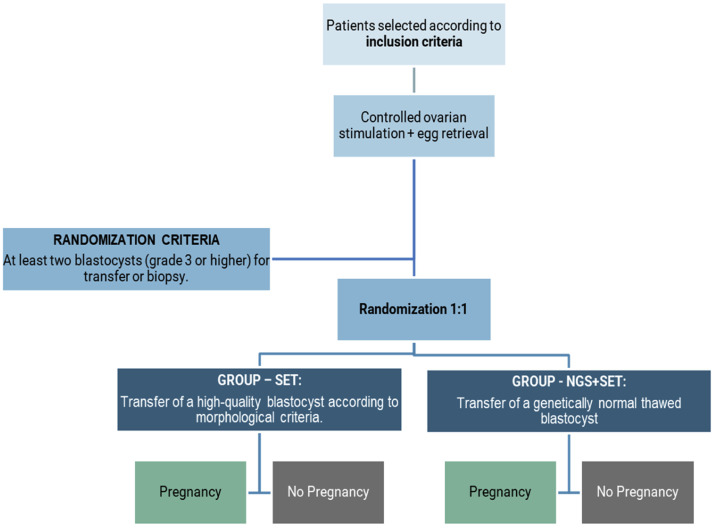
Representative flowchart of the study.

After the evaluation of patients and selection based on inclusion and exclusion criteria, patients underwent controlled ovarian stimulation and egg retrieval. Embryonic classification was performed after culture until the blastocyst stage, and then randomization was carried out for patients with at least two available good-quality embryos. Patients who did not have the formation of at least two good-quality embryos were not randomized. The randomization was conducted at a 1:1 ratio using a computerized program in a Microsoft Excel® table. Thus, two study groups were formed, as described below and represented in Fig. 1.

SET Group: Women subjected to the elective transfer of a single good-quality embryo according to morphological criteria.^26^

NGS + SET Group: Women whose embryos were biopsied and underwent elective transfer of a single euploid (genetically normal) embryo based on NGS results.

### Controlled ovarian stimulation and embryo culture

A comprehensive medical history and routine diagnostic tests for women, such as transvaginal ultrasound, hysterosalpingography, and baseline hormonal assays including Follicle-Stimulating Hormone (FSH), Luteinizing Hormone (LH), and Estradiol (E2) from the 1st to the 3rd day of the menstrual cycle, as well as Thyroid-Stimulating Hormone (TSH) and thyroxine T4 levels, were performed. Seminal analysis for the male partner and mandatory serological tests for the couple were also conducted. Following the conjugal evaluation and analysis of the tests, controlled ovarian stimulation was scheduled for in vitro fertilization treatment.

For controlled ovarian stimulation, patients received 225 IU/day of recombinant gonadotropin from the third day of the menstrual cycle for three days, followed by 150 IU/day for an additional seven days. Serial transvaginal ultrasounds were performed on the 6th, 8th, and 11th days of ovarian stimulation to monitor follicular development, and gonadotropin doses were adjusted according to ovarian response when necessary. Final follicular maturation was induced by administering a Gonadotropin-Releasing Hormone (GnRH) analog on the 11th day of stimulation, and follicular puncture was performed 36 h after GnRH analog administration.

### Embryo culture and embryo classification

After oocyte retrieval, oocytes were denuded and fertilized by Intracytoplasmic Sperm Injection (ICSI), followed by culture in a triple gas incubator (90 % N_2_, 5 % O_2_, and 6 % CO_2_) at 37 °C. Fertilization was assessed 18 h after ICSI, with normal fertilization identified by the presence of two Pronuclei (2PN). Embryos were evaluated daily under an inverted microscope until the third day of development, considering (i) Number of blastomeres, (ii) Percentage of fragmentation, (iii) Symmetry, (iv) Multinucleation, and (v) Defects in the cytoplasm and zona pellucida. On day-5, blastocysts were evaluated for blastocele expansion, number, and cellular organization of the Inner Cell Mass (ICM) and trophectoderm, according to Gardner et al.^26^ A blastocyst of good quality was considered adequately expanded (Grades-3, -4, or -5), with ICM Grades A or B, and trophectoderm Grades A or B.

### Embryo biopsy

Embryo biopsy was performed on the 5th or 6th day of development, at the blastocyst stage, using an inverted microscope and a Hoffman optical objective with 40× magnification and 2 micropipettes adapted to the micromanipulator: one for fixing the embryo and the other for aspirating cells from the trophectoderm. After biopsy, embryos were removed from the biopsy plate and placed back in the culture plate, then cryopreserved using the previously described vitrification technique. The extracted cells were transferred to a microtube containing 2.5 microliters of Saline in Phosphate Buffer (PBS). The embryonic trophectoderm cells were sent for analysis in a reference laboratory.

### Next-Generation sequencing (NGS)

NGS was performed in a reference laboratory according to established standard methods. The cell sample obtained from the blastocyst trophectoderm was subjected to DNA lysis and amplification using the Whole Genome Amplification (WGA) technique. This is a robust method for amplifying a complete genome, starting from minimal concentrations (nanograms) of DNA, resulting in amplified products.

The analysis of results was carried out through appropriate bioinformatics software, where the sample result was compared with the hg19 reference genome. Pattern detection and classifications were determined by Copy Number Variation (CNV) values, such as euploid (between 1.80 and 2.20), pure aneuploid (<1.20 or >2.80), and mosaic (between 1.20 and 1.80 or between 2.20 and 2.80).

### Embryo transfer

Embryo transfer was performed for each group according to randomization. For groups that did not undergo NGS (SET Group), only the morphological classification previously described was used. For the NGS+SET group, the embryo was selected based on the NGS result, and only euploid embryos were transferred.

Embryo Transfer (ET) was performed after endometrial preparation using 100 µg of transdermal estradiol daily from the third day of the menstrual cycle. Patients underwent serial transvaginal ultrasounds on the 7th and 12th days of endometrial preparation to monitor endometrial development, and 600 µg of micronized progesterone were added vaginally for five days once the endometrium reached 7 mm with a trilaminar appearance observed on ultrasound. The transfer was performed 5-days after the introduction of progesterone. After the procedure, 600 µg of micronized progesterone were maintained vaginally, and 100 µg of transdermal estradiol were continued daily until the diagnosis of pregnancy by serum beta-hCG measurement.

The transferred embryos were thawed, assessed for survival, and then transferred to the uterine cavity of the patients according to the standard protocol. Pregnancy was diagnosed by beta-hCG measurement nine days after embryo transfer, and for positive cases, it was confirmed by ultrasound at six weeks of gestation, observing the number of gestational sacs, number of embryos, and fetal heartbeat.

### Sample size

To calculate the sample size based on the initial data of this study, the equivalence test for binary outcomes was used, considering the rate of ongoing pregnancy per transferred cycle. An ongoing pregnancy rate of 60 % for both groups and an equivalence limit of 20 % were considered, with an alpha error (p) of 5 % and a beta error (power) of 80 %. To show or not show a difference between the standard treatment (SET Group) and the experimental group (NGS + SET Group) with a 60 % ongoing pregnancy rate in both groups, it was necessary to include 206 patients (103 in each group) to have 80 % certainty that the limits of a bilateral 90 %.

### Statistical analysis

Each couple has a record containing clinical and laboratory data obtained during the assisted reproduction cycle, as well as gestational data for patients with a positive pregnancy. Such information was tabulated and analyzed along with the results of this study.

The analyses were conducted using the statistical software Jamovi. Continuous numerical variables are presented as “mean ± standard deviation” and minimum and maximum values, while categorical variables are presented as frequency and percentages (%). Numerical variables were compared using Student's *t*-test, and categorical variables were compared using Pearson's Chi-Square test. Values of *p* < 0.05 were considered statistically significant, following international standards for the interpretation of biostatistical data. Descriptive and inferential analyses were performed to compare the two study groups, referred to as the SET group and the NGS + SET group.

## Results

### Descriptive analysis

**Fig. 2:**
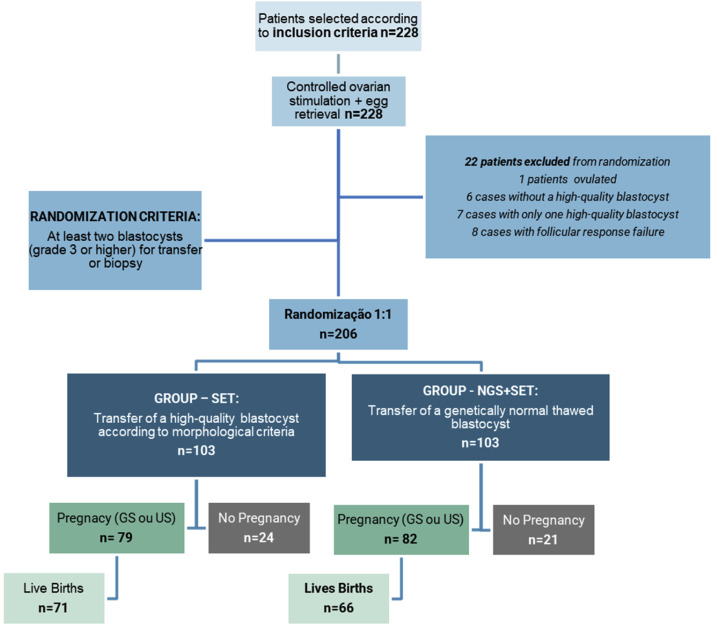
Flowchart representing the descriptive result of the study.

Initially, between the years 2019 and 2021, 228 patients were included to initiate in vitro fertilization treatment. From this initial number, 22 patients (9.6 %) were excluded from randomization selection. One patient (4.5 %) was excluded due to ovulation, 6 patients (27.3 %) were not included because they did not produce any blastocyst of good quality, 7 patients (31.8 %) had the formation of only 1 good-quality blastocyst, and 8 patients (36.4 %) had their cycle canceled during the ovarian stimulation phase due to follicular response failure (Fig. 2).

### General characteristics of the study group

Demographic and clinical data of the treatment cycles were analyzed, including patients not selected for randomization, to verify if the profile of those excluded was the same as that of the randomized ones, ensuring that their exclusion does not introduce biases.

When analyzing all patients included for the start of treatment, the groups showed homogeneity regarding age, Body Mass Index (BMI), basal FSH levels, and antral follicle count, as shown in the data described in Table 1. Regarding the duration of infertility, a difference was observed between the groups (F (2;52.9) = 12.24, *p* = 0.007). The Games-Howell post-hoc test for heterogeneous variances showed that patients excluded from randomization had a significantly longer duration of infertility compared to the SET (*p* = 0.009) and NGS + SET groups (*p* = 0.018). The FSH dose administered to the excluded randomization group was significantly lower (*p* < 0.001) than the randomized groups, which can be explained by the cancellation of 8 patients due to stimulation failure detected at the first ultrasound check. In all groups, cycles were conducted using recombinant gonadotropin and agonist as a trigger.

**Table 1:** Demographic data and baseline clinical characteristics of all patients included in the study.

	Set (*n* = 103)		NGS + SET (*n* = 103)		Excl. randomization (*n* = 22)		p^a^
	Mean ± SD	Min‒Max	Mean ± SD	Min‒Max	Mean ± SD	Min‒Max	
**Age (years)**	33.7 ± 3.0	24.5‒37.5	33.6 ± 2.9	26.2‒37.1	33.6 ± 3.2	24.1‒37.3	0.938
**Infertility duration (months)**	38.0 ± 27.6	12.0‒120	41.6 ± 28.8	12‒120	79.3 ± 57.9	12‒236	**0.007**
**BMI (kg/m^2^)**	23.7 ± 3.26	17.6‒30.8	24.1 ± 3.32	16.9‒32.9	24.6 ± 2.90	19.7‒31.2	0.377
**Basal FSH**	5.40 ± 2.08	0.200‒11.0	6.05±2.33	2.00‒19.0	5.63 ± 2.19	2.10‒8.80	0.114
**Antral follicle count**	18.0 ± 5.77	7.00‒33.0	17.9 ± 8.02	9.00‒70.0	13.9 ± 7.57	8.00‒35.0	0.059
**FSH dose (IU)**	**1980±247**	**1500‒3150**	**1909±223**	**1500–2625**	**1383±606**	**900±2100**	**<0.001**

a As variáveis numéricas foram comparadas por One-way ANOVA.

The demographic and clinical data of the treatment cycles for the randomized patients are described in Table 2. The groups were homogeneous regarding age at the beginning of the procedure (*p* = 0.721), duration of infertility (*p* = 0.630), BMI (*p* = 0.395), and antral follicle count (*p* = 0.736). Regarding basal FSH, the authors observed that patients in the NGS + SET group had a slightly higher basal FSH than the control group (*p* = 0.034). Concerning the controlled ovarian stimulation outcome, the dose of administered FSH in the NGS + SET group was significantly lower (*p* < 0.033) than in the control group.

**Table 2:** Demographic profile and baseline clinical characteristics of the patients included in the study.

	Set (*n* = 103)		NGS + SET(*n* = 103)		
	Mean ± SD	Min‒Max	Mean±SD	Min‒Max	p^a^
**Age (years)**	33.7 ± 3.0	24.5‒37.5	33.6 ± 2.9	26.2‒37.1	0.721
**Infertility duration (months)**	38.0 ± 27.6	12.0‒120	41.6 ± 28.8	12‒120	0.360
**BMI (kg/m^2^)**	23.7 ± 3.26	17.6‒30.8	24.1 ± 3.32	16.9‒32.9	0.395
**Basal FSH**	5.40 ± 2.08	0.200‒11.0	6.05 ± 2.33	2.00‒19.0	**0.034**
**Antral follicle count**	18.0 ± 5.77	7.00‒33.0	17.9 ± 8.02	9.00‒70.0	0.736
**FSH dose (IU)**	1980 ± 247	1500‒3150	1909 ± 223	1500‒2625	**0.033**

a Numeric variables were compared using Student's *t*-test.

The demographic profile and baseline clinical data of the partners of the included patients in the study were homogeneous among the groups, as described in Table 3. There was no significant difference regarding age, sperm concentration, and the presence of male factor.

**Table 3:** Demographic profile and baseline clinical characteristics of the partners of the patients included in the study.

	SET (*n* = 103)		SET + NGS (*n* = 103)		Excl. randomization (*n* = 22)		
	Mean ± DP ou n (%)	Min‒Max	Mean ± DP ou n (%)	Min‒Max	Mean ± DP ou n (%)	Min‒Max	p^a^
**Age (years)**	37 ± 5.62	24.9 ‒62.5	35.9 ± 4.55	25‒53	36.4 ± 7.71	23‒54	0.300
**Sptz concentration (mi/mL)**	52.6 ± 56.4	5.5‒276	49 ± 33.5	5‒150	33.4 ± 30	5.6‒90	0.061
**Male factor**	39 (37.9 %)		28 (27.2 %)		22 (100 %)		0.070

a Numeric variables were compared using One-way ANOVA, and categorical variables were compared using the Chi-Square test.

**Fig. 3:**
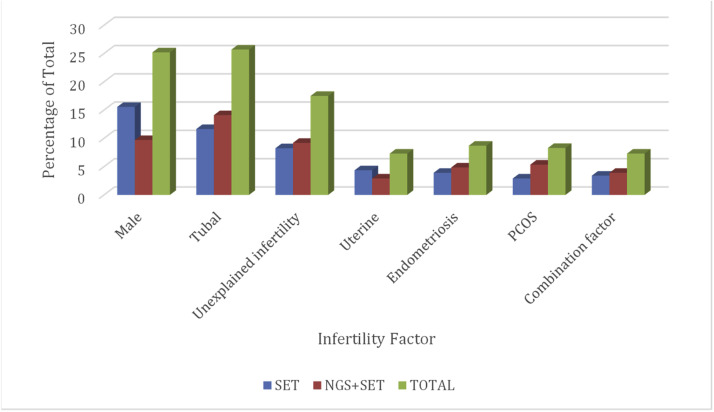
Chart of the frequency of infertility factor in the study groups.

The patients included in the study were categorized according to infertility factors classified by the Society for Assisted Reproductive Medicine (SART). The frequency of infertility factors and comparison between the SET and NGS + SET groups are presented in Fig. 3. There was no statistical difference between the groups, and categorical variables were compared using the Chi-Square test.

### Laboratory results

Regarding the laboratory outcomes of the SET and NGS + SET groups, the results were similar, with no statistical differences in oocyte characteristics. However, the blastulation rate and the average number of formed and frozen blastocysts were higher in the SET group. The higher number of frozen embryos in the SET group was expected since they were not subjected to biopsy (Table 4).

**Table 4:** Laboratory outcomes of the randomized groups' patients.

	SET (*n* = 103)	NGS + SET (*n* = 103)	p^a^
	Mean ± DP	Mean ± DP	
**N° of oocytes retrieved**	18.7 ± 9.01	17.2 ± 9.58	0.266
**N° of MII oocytes retrieved**	14.6 ± 6.95	13.1 ± 6.16	0.106
**Fertilization rate**^b^	77.5 %	81.1 %	0.278
**Blastulation rate**^c^	68.8 %	61.1 %	**0.002**
**N° of blastocyst**	8.86 ± 3.37	7.59 ± 3.99	**0.020**
**N° of good-quality blastocysts**	6.98 ± 2.95	6.05 ± 3.07	**0.027**
**N° of frozen blastocysts**	6.96 ± 2.94	4.17 ± 1.84	**<0.001**
**N° of biopsied embryos**	‒	5.88 ± 2.61	‒
**N° euploides embryos**	‒	3.99 ± 1.75	
**Euploidy blastocysts rate**	‒	69.1 %	‒

a Numeric variables were compared using Student's *t*-test, and categorical variables were compared using the Chi-Square test.

b Normal fertilization rate calculated by the number of fertilized 2PN divided by the number of injected eggs.

c Blastulation rate calculated by the number of formed blastocysts divided by the number of injected eggs.

### Clinical results in study groups after the first embryo transfer

**Fig. 4:**
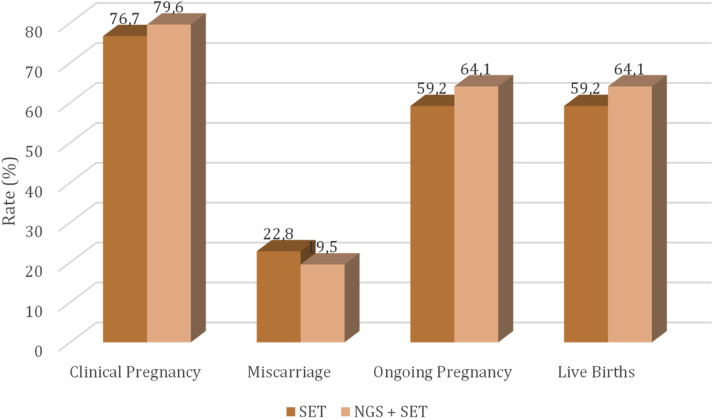
Total pregnancy, total miscarriage, ongoing pregnancy, and live birth rates of the study groups.

Clinical outcome data after the first embryo transfer are described in Table 5 and depicted in Fig. 4. All cycles in the NGS group had at least one euploid embryo; hence, there was no cancellation of embryo transfer due to the absence of a euploid embryo in this study group. Similarly, there was no cancellation of embryo transfer due to the absence of a good-quality embryo in the SET group. All transfers performed were of a single embryo, and most transferred embryos were on the fifth day of culture (D5) in both groups (SET 90 % and NGS + SET 92 %; *p* = 0.874). There was no statistical difference between the groups regarding the pregnancy rate, abortion rate, ongoing pregnancy rate, or live birth rate. One ectopic pregnancy was included in the total abortion group, and one diamniotic monochorionic twin pregnancy occurred in the NGS + SET group (0.01 %).

**Table 5:** Clinical results after the first embryo transfer.

Clinical outcome per transferred cycle	SET (*n* = 103)	bNGS + SET (*n* = 103)	p^a^
	n (%)	n (%)	
Total pregnancy	79/103 (76.7 %)	82/103 (79.6 %)	0.613
Pregnancy loss	18/79 (22.8 %)^c^	16/82 (19.5 %)	0.611
Ongoing pregnancy (>12 w)	61 (59.2 %)	66 (64.1 %)	0.474
live birth rate	61 (59.2 %)	66 (64.1 %)	0.474

a Categorical variables were compared using the Chi-Square test.

b Multiple gestation was observed in the NGS + SET group.

c Ectopic pregnancy was observed in the SET group, which was recorded as a miscarriage.

## Discussion

Traditionally, morphology-based classification has been the primary technique used In Vitro Fertilization (IVF) to assess and select the most competent embryo for transfer.^26^ Technologies have been developed in the fields of genomics, transcriptomics, proteomics, metabolomics, and time-lapse imaging to try to assist and improve embryo selection. However, the focus has been on the analysis of the copy number of the 24 chromosomes for the evaluation and transfer of embryos diagnosed as euploid, also known as Preimplantation Genetic Testing for Aneuploidy (PGT-A).^7^^,^^8^ Various molecular techniques have been used during IVF cycles to determine ploidy, including Fluorescence In Situ Hybridization (FISH), Comparative Genomic Hybridization (CGH), array CGH (aCGH), digital Polymerase Chain Reaction (dPCR), Single Nucleotide Polymorphism (SNP), quantitative real-time Polymerase Chain Reaction (qPCR), and Next-Generation Sequencing (NGS). These technologies vary in terms of cost and time for completion, and only a few of these methods allow the transfer of fresh embryos.

An important concept, classically applied to the emergence of new technologies in various research fields, is the “Gartner Hype Cycle” theory.^27^^,^^28^ These concepts suggest that the use of PGT-A in IVF cycles is still entering the plateau phase of its productivity. There are some well-established indications, but there are still gaps in evidence regarding the benefits of the indication in all cases of infertility or, at least, in those infertile couples with a good prognosis.

The early iterations of PGT-A evaluated a subset of chromosomes, mainly using FISH to examine 5 to 10 individual chromosomes. Despite the hypothesis that transferring only euploid embryos should improve IVF outcomes, many studies with this initial approach failed to demonstrate benefits.^11^^,^^29^ Since 24-chromosome techniques became available, there have been few robust studies that have shown clear scientific evidence on the success rates of in vitro fertilization treatment with preimplantation genetic diagnosis.

The indication of NGS technology for preimplantation genetic testing for aneuploidy in all IVF treatments is not yet supported by current literature. Studies cited in this work already point to a lack of evidence, especially in cases of infertile couples with a good prognosis.^19^^,^^29^

In 2021, Sarkar et al., in a retrospective study, analyzed over 56.000 IVF cycles in more than six thousand patients with the aim of evaluating whether preimplantation genetic analysis is associated with a decrease in the rate of early pregnancy losses.^30^ When comparing the groups that underwent PGT-A with the group that did not undergo embryo biopsy, there was no statistically significant difference in live birth rates and abortion rates for any age group analyzed. In a meta-analysis with 9 clinical trials, Cheng et al., 2022, with an evaluation of a total of 3334 patients, found no difference in success rates between the group that underwent PGT-A and the group that did not undergo genetic testing.^31^ The only statistically significant difference was observed in live birth rates in the subgroup of older women (above 38-years according to this study's criteria).

Analyzing specific percentages such as cumulative pregnancy rate, live birth rate, and success rate per embryo transfer in cases of IVF with PGT-A, some conflicting results in the literature regarding the use and indications of preimplantation genetic embryo analysis are observed.^32^ Some studies show a relative increase in the pregnancy rate per embryo transfer, but there is no increase in live birth rates or cumulative pregnancy rates.^19^^,^^33^

Amid various doubts, some groups reinterpreted the “Star” study published by Munne et al. in 2019. In 2020, Pagliardini et al., led one of these groups and raised important observations and questions.^34^ After reinterpretation, it was suggested that PGT-A did not significantly increase live birth rates per embryo transfer or the intention-to-treat in women aged 25 to 40. Moreover, it was estimated that the transfer of only euploid blastocysts after PGT-A led to a drop in live birth rates from 82.2 to 50 % for competent embryos, suggesting embryonic waste.

In 2023, Kucherov et al., in a retrospective cohort study, analyzed success rates of over 130.000 IVF cycles.^35^ The results showed that the use of PGT-A significantly decreased live birth rates, except in the group of patients over 40-years old. As additional information, it emphasized that this decrease was more pronounced in the subgroup of patients under 35-years old.

The conflicting data in the literature and the lack of more robust evidence have raised questions about the use and indications of preimplantation genetic analysis in IVF treatment, especially regarding the failures of the technology itself and a possible deleterious relationship due to embryo injury from the biopsy procedure for genetic analysis.^34^ Additionally, there are doubts regarding the potential waste of embryos during the IVF process with the use of PGT-A, especially concerning mosaicism.^36^ Some studies suggest that there are physiological mechanisms for correcting genetic errors in embryos throughout their development and mechanisms related to embryonic plasticity in the pre- and post-implantation phases.^36^^,^^37^

It is a reality in the global literature that studies evaluate the transfer of embryos with some types of mosaicism. In 2021, Viotti et al., analyzed success rates and showed that euploid embryos have higher success rates.^38^ However, after performing a thousand mosaic transfers, it was shown that embryos with different degrees of mosaicism have the potential to generate pregnancy and live births. These data suggest that throughout development, genetic changes in some mosaic embryos can be physiologically corrected and give rise to healthy live births.^39^^,^^40^

One of the common indications for PGT-A in In Vitro Fertilization (IVF) cycles is recurrent miscarriage. Some studies suggest that genetic analysis would select euploid embryos, increase the pregnancy rate, reduce the time to conceive, and decrease costs associated with eventual procedures and post-abortion follow-up, as well as repeated in vitro fertilization cycles.^23^^,^^24^^,^^41^ In 2015, Murugappan et al. compared the cost-effectiveness of recommending genetic analysis for aneuploidies in cases of recurrent miscarriage.^42^ After results, this study showed that the cost for IVF with PGT-A was over 100 times higher than treatment without embryo biopsy and suggests that the use of PGT-A is not beneficial in reducing the cost for infertile couples to have healthy live-born children.

The cost-effectiveness and economic analysis are very important for public health policy, where human reproduction services are funded by the government, as is the case in our center.

More recently, in 2023, He et al., in a study with the Chinese population, with women aged between 20 and 37, also showed that the indication of preimplantation genetic diagnosis is not cost-effective in cases of recurrent miscarriage.^43^ Even with the need for more transfers, the group that underwent IVF treatment without biopsy had a lower cost per live birth.

The sample size was calculated considering an ongoing pregnancy rate of 60 %. To show or not show a difference between the standard treatment (SET Group) and the experimental group (NGS + SET Group) with a 60 % ongoing pregnancy rate in both groups, it was necessary to include 206 patients (103 in each group) to have 80 % certainty that the limits of a bilateral 90 %. The equivalence limit of 20 % can be considered a limit of the study, but it was used to make the study feasible in relation to the sample size. However, the recalculated power after the results was actually 80 %.

The clinical data from the present study, such as the number of collected oocytes, number of MII oocytes, normal fertilization rate, and number of blastocysts, did not show significant differences between the groups. The same occurred in the evaluation of infertility factors, which, despite presenting slightly different percentages between the SET and NGS + SET groups, had no statistically significant impact on the results. Basal FSH was higher in the NGS + SET group (*p* = 0.034). However, the primary outcome was the pregnancy rate. Since the pregnancy rate is closely related to the female age and not to the duration of infertility or normal variations in FSH. One of the inclusion criteria was that FSH had to be less than 12 mIU/mL, but the difference between the groups would not influence the result. The dose of FSH administered to the NGS + SET group was significantly lower (*p* < 0.033) than in the SET group. Nonetheless, as the gonadotropin dose is adjusted according to the patient's response, aiming to collect an adequate number of oocytes, the stimulation result was similar between the groups, as evidenced by the number of oocytes collected, which was similar, making the difference in the administered gonadotropin dose clinically insignificant.

Additionally, blastulation rates, high-quality blastocysts, and the average number of formed and frozen blastocysts were higher in the SET group. This difference may be regarding the failures of the technology itself and a possible deleterious relationship due to embryo injury from the biopsy procedure for genetic analysis.^34^ The higher number of frozen embryos in the SET group was expected since they were not subjected to biopsy. As data were analyzed only after the first embryo transfer, these differences did not have clinical significance in this study. In addition, a limitation of the study was not powered to detect differences in secondary outcomes.

Despite this study having a limitation regarding the sample size of the groups, with the laboratory data obtained, the authors demonstrate that, for couples with a good prognosis, with women up to 37-years-old, the clinical pregnancy rate of the NGS + SET group did not show a statistical difference when compared to the SET group after the first embryo transfer. The same occurred with total abortion rates and live birth rates, which were equivalent between the studied groups. Additionally, the euploidy rate of the NGS + SET group was 69.1 %, following current literature data for patients with a good prognosis.^44^

## Conclusion

This clinical trial suggests that the embryo genetic analysis in IVF treatments for infertile couples with a good prognosis does not increase clinical pregnancy and live birth rates.

## Declarations

ClinicalTrials.gov Identifier: NCT03758833.

ClinicalTrials.gov date of registration: November 29, 2018.

Ethics approval: Clinical trial conducted with patient clinical data and manipulation of human embryos. Therefore, previously authorized by an appropriate ethics committee, and the Informed consent was obtained from all individual participants included in the study.

(CAPPesq): 2.226.398

Comitê de Ética em Pesquisa (CEP) CONEP (number 4.567.830).

## Authors’ contributions

PFMP and PAAM conceived and designed the study, contributed to data collection, and wrote the final manuscript. MSN, CAZN and PF assisted with data collection. PFMP wrote the final manuscript. MLM, JMSJ, PAAM and ECB assisted with study design. All authors contributed to the interpretation of the data and writing of the final article, including the discussion and conclusions.

## Funding

This study was conducted at a government-funded institution, and the authors did not receive support from any organization for the submitted work.

## Data availability statement

The datasets generated and/or analyzed during the current study are available from the corresponding author upon reasonable request.

## Conflicts of interest

This study was conducted at a government-funded institution. The authors declare no competing interests.

## Appendix Supplementary materials

Image, application 1

Image, application 2
