# Supplementary figures and images for: Elective single embryo transfer in in vitro fertilization cycles with or without preimplantation genetic testing using next-generation sequencing: A randomized clinical trial
Source: Clinics (Sao Paulo). 2026 Feb 24;81:100870. doi: 10.1016/j.clinsp.2026.100870 (PMC12973516; doi:10.1016/j.clinsp.2026.100870)

FLOWCHART – CLINICAL TRIALS


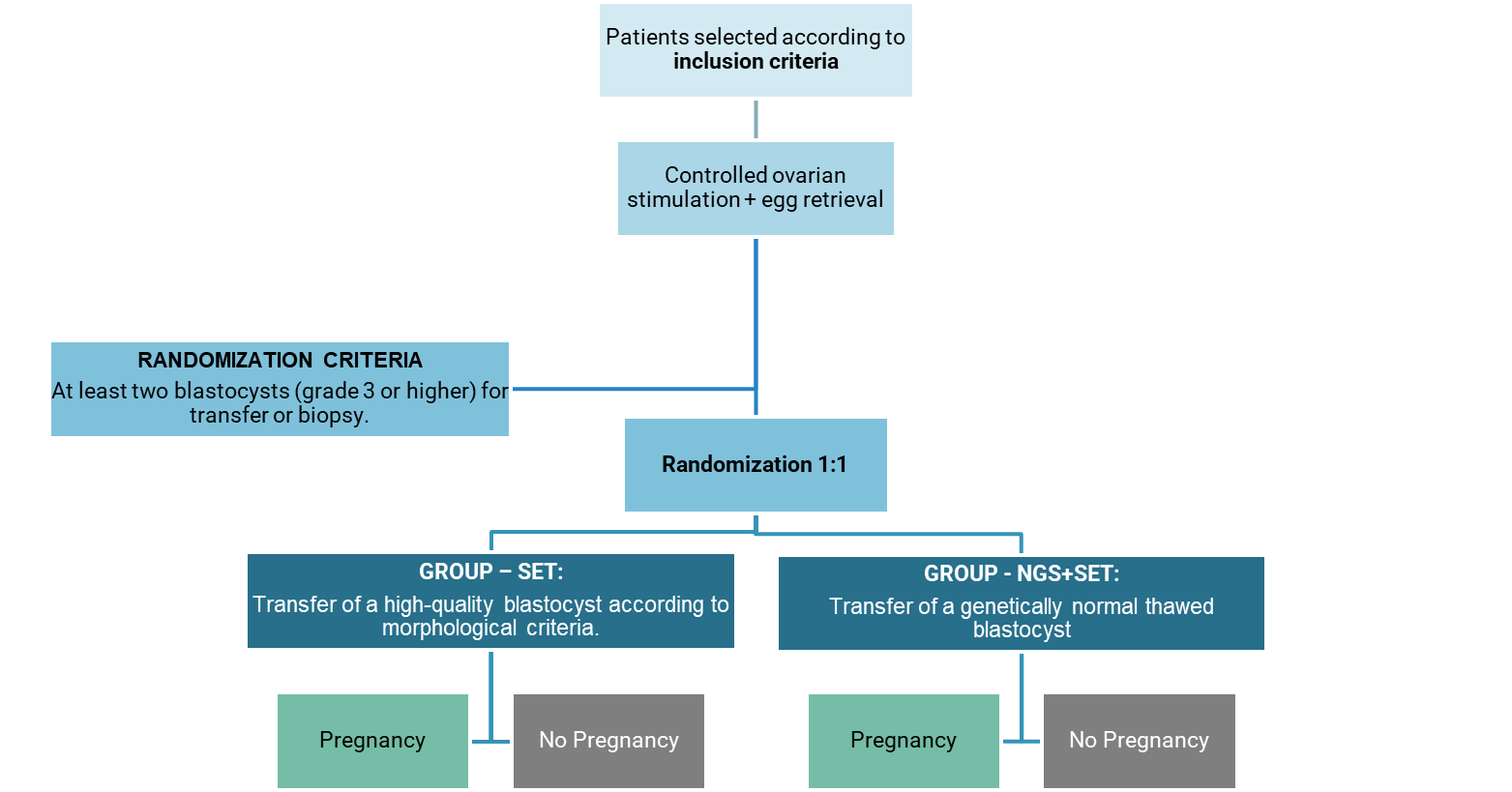

Supplement: Supplementary file 2 [file mmc2.docx]
